# Specimen-displacement correction for powder X-ray diffraction in Debye–Scherrer geometry with a flat area detector

**DOI:** 10.1107/S1600576722011360

**Published:** 2023-02-01

**Authors:** Benjamin S. Hulbert, Waltraud M. Kriven

**Affiliations:** a Materials Science and Engineering, University of Illinois at Urbana-Champaign, 1304 W. Green St., Urbana, Illinois 61801, USA; Australian Synchrotron, ANSTO, Australia

**Keywords:** Debye–Scherrer, transmission, speci­men-to-detector distance, displacement correction equation, powder X-ray diffraction, area detectors

## Abstract

The effect of small changes in the speci­men-to-detector distance on the unit-cell parameters is examined for synchrotron powder diffraction in Debye–Scherrer (transmission) geometry with a flat area detector. An analytical correction equation is proposed to fix the shift in 2θ values due to speci­men capillary displacement. This equation does not require the use of an internal reference material, is applied during the Rietveld refinement step, and is analogous to the speci­men-displacement correction equations for Bragg–Brentano and curved-detector Debye–Scherrer geometry experiments, but has a different functional form. The 2θ correction equation is compared with another speci­men-displacement correction based on the use of an internal reference material in which new integration and calibration parameters of area-detector images are determined. Example data sets showing the effect of a 3.3 mm speci­men displacement on the unit-cell parameters for 25°C CeO_2_, including both types of displacement correction, are described. These experiments were performed at powder X-ray diffraction beamlines at the National Synchrotron Light Source II at Brookhaven National Laboratory and the Advanced Photon Source at Argonne National Laboratory.

## Abstract 2

An analytical correction equation is proposed to fix the shift in 2θ values caused by speci­men capillary displacements for powder diffraction experiments in Debye–Scherrer (transmission) geometry with a flat area detector. The accuracy of this equation and the corresponding corrections were verified by comparing it with a correction based on new integration parameters from an internal reference material.

## 1. Introduction

Powder X-ray diffraction (XRD) is an important characterization technique in many disciplines and industries (Margiolaki *et al.*, 2019 ▸; Cernik & Barnes, 1995 ▸; Harlow, 1999 ▸). Since the origins of laboratory diffractometers, synchrotrons and free electron lasers (FELs), there have been frequent advances in technology, including more advanced detectors that have enabled rapid and improved data collection. The increasing accessibility of user facilities, in part through mail-in experiments, has made the advantages of synchrotron and FEL sources available to a growing number of crystallographers. It is important with these advances that related analytical techniques be shared for accurate and efficient data processing. The motivation for this study is the development of a speci­men-displacement correction equation that can be implemented easily in existing Rietveld refinement software for an experimental geometry that is common at powder diffraction synchrotron beamlines.

Specimen-displacement errors in angular-dispersive powder XRD have been mitigated with the use of analyzer crystals, rotation of the speci­men capillary, measurements at multiple detector distances, the addition of an internal reference material (RM) and the use of 2θ correction equations for cer­tain geometries. Specimen capillaries are often centered with a multi-axis goniometer stage, with directions of translation and tilt adjusted in order to accurately control the relative positions of the X-ray source, speci­men and detector. Alignment in the direction perpendicular to the X-ray beam is easier than parallel alignment because the capillary can be scanned through the X-ray beam and a plot of intensity as a function of position can show absorption from the speci­men to give the center location of the X-rays.

Even with careful capillary alignment there can be variation in the position of subsequent speci­men capillaries. Placing an analyzer crystal between the speci­men and the detector allows only the elastic reflection of X-rays that meet the Bragg condition of the analyzer crystal, which also eliminates background intensity due to fluorescence and inelastic scattering (Fauth *et al.*, 2000 ▸; Hastings *et al.*, 1984 ▸). An analyzer crystal eliminates the speci­men-displacement error and has high angular resolution, but only measures one angle at a time, so it must scan through the 2θ range being studied (Cernik & Barnes, 1995 ▸). Multiple detectors and analyzer crystals in parallel can speed up data collection, but they still require time to scan through a subset of the 2θ angles (Hodeau *et al.*, 1998 ▸).

**Figure 1:**
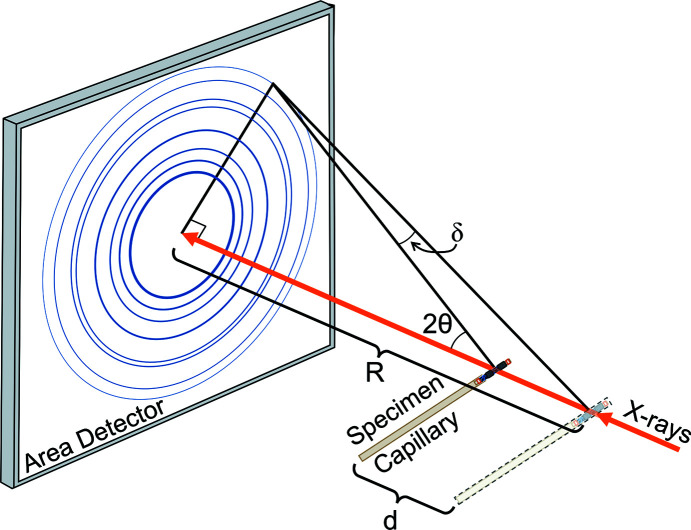
Diagram of a Debye–Scherrer (transmission) powder XRD experiment, showing the effect of speci­men displacement on the 2θ value for a flat area detector. This type of experimental setup with an area detector is common at synchrotron beamlines.

Large area detectors allow for fast data collection, as well as measurements of texture, stress and crystallite size from a full powder XRD pattern collected with a single exposure, without scanning through each angle (Norby, 1997 ▸). Rapid data collection allows for *in situ* or *operando* experiments, in which time-sensitive measurements are important. For example, chemical reactions or phase-change dynamics can be measured when XRD patterns can be collected quickly or continuously. However, these area detectors cannot be used with analyzer crystals and can have speci­men-displacement errors, as shown in Fig. 1 ▸. Specimen-displacement errors are regularly cor­rected by rotating the speci­men capillary during data collection with a capillary spinner. A capillary spinner keeps the average position of the capillary at the center of rotation, so if the capillary is not perfectly centered in the goniometer there will be a small increase of peak widths (Stock *et al.*, 2019 ▸) in the diffraction pattern instead of a peak shift error. Additionally, speci­men spinners increase sampling statistics (Ida *et al.*, 2009 ▸) because more powder particles meet the Bragg condition for diffraction during an XRD scan.

Calibration and integration parameters for an area detector are usually found from a reference material (RM) like CeO_2_, LaB_6_, Si, Al_2_O_3_, NaCl, Pt or Ni, which is measured separately (externally) from the speci­men materials (Cline *et al.*, 2019 ▸). This procedure can be completed using *GSAS-II* (Toby & Von Dreele, 2013 ▸), *FIT2D* (Hammersley *et al.*, 1995 ▸; Hammersley, 2016 ▸) or a similar program to arrive at the intensity versus 2θ powder diffraction pattern. These parameters are then used to azimuthally integrate all area-detector images for an experiment (Cline, 1999 ▸; Cervellino *et al.*, 2006 ▸), with the assumption that the speci­men position and other parameters do not change throughout a synchrotron experiment, which may not be true (Andersen *et al.*, 2018 ▸). If there is movement of the goniometer or detector, a speci­men spinner is not used, or if other calibration and integration parameters change through­out a synchrotron experiment, then a correction based on the use of an internal RM mixed with each speci­men can be employed. This follows the same calibration procedure as for an external RM, in which 2D area-detector images are used for image processing, followed by integration, except it is completed for each speci­men capillary. A related method can simultaneously determine the speci­men-to-detector distance and the X-ray energy on the basis of two or more XRD patterns collected with a measured detector shift (Hart *et al.*, 2013 ▸; Horn *et al.*, 2019 ▸). These techniques would not be possible if only the intensity versus 2θ XRD pattern was available.

**Figure 2:**
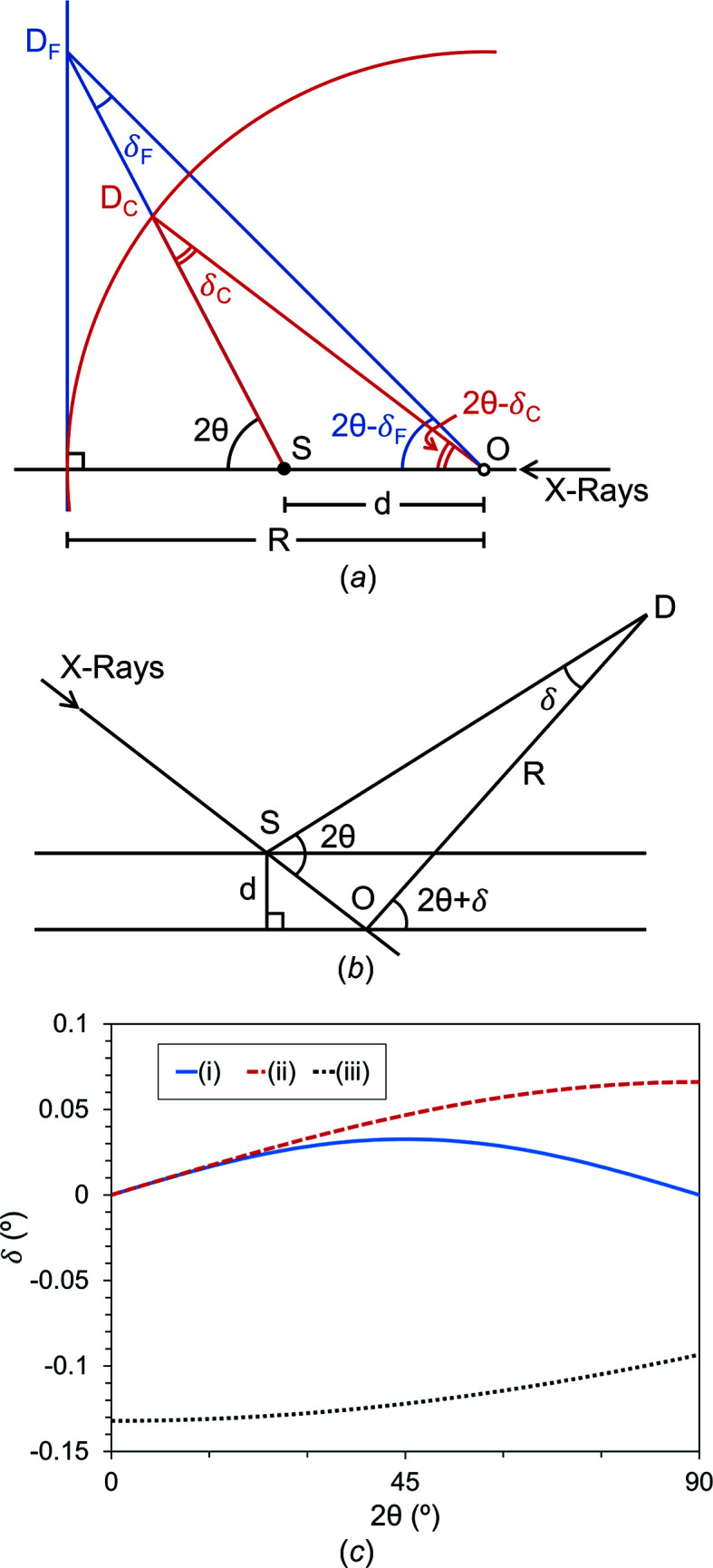
Comparison of the 2θ corrections (δ) for speci­men displacements in three experimental geometries, including (*a*) Debye–Scherrer with a flat detector (derived in this article) in blue with subscripts F, Debye–Scherrer with a curved detector in red (Gozzo *et al.*, 2010 ▸; McCusker *et al.*, 1999 ▸) with subscripts C and (*b*) Bragg–Brentano (McCusker *et al.*, 1999 ▸). The speci­men capillary is at S, the center of the goniometer is at O, a length *R* from the detector, and the diffracted ray hits the detector at D. (*c*) Plots of the functional form of each 2θ correction for (i) Debye–Scherrer with a flat detector, (ii) Debye–Scherrer with a curved detector and (iii) Bragg–Brentano, where *R* = 173.5 mm and *d* = 0.2 mm for each. There is no displacement in the vertical direction, *d*_⊥_ = 0 mm, for (ii).

The use of an internal RM is not always possible due to reaction with the speci­men material or an overlap of the Bragg peaks with the speci­men being studied, in which case a correction equation can apply a shift to the 2θ values in an intensity versus 2θ diffraction pattern. This type of correction is used during the Rietveld refinement step (Rietveld, 1969 ▸; Loopstra & Rietveld, 1969 ▸). There is an equation for a corrective shift, δ, in 2θ angle to compensate for a sample displacement, *d*, from the goniometer center for a speci­men-to-detector distance, *R*, in Debye–Scherrer geometry with a curved area detector (Gozzo *et al.*, 2010 ▸; Pramanick *et al.*, 2009 ▸; McCusker *et al.*, 1999 ▸), *i.e.*


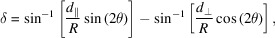

where *d*_∥_ and *d*_⊥_ denote displacements parallel and perpendicular to the X-ray direction, respectively. There is also a correction for Bragg–Brentano (reflection) geometry (McCusker *et al.*, 1999 ▸) given by 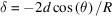 . To date, no correction equation has been published for a flat area detector in the Debye–Scherrer geometry shown in Fig. 1 ▸, even though it is common in synchrotron powder XRD experiments. Comparisons of these 2θ correction equations for speci­men displacements and their experimental geometries are shown in Fig. 2 ▸. The main goals for this article were to propose a speci­men-displacement 2θ correction equation for Debye–Scherrer geometry with a flat area detector, to demonstrate the correction of a speci­men displacement with an internal RM, and to evaluate the effectiveness of the 2θ correction equation when compared with the correction from an internal RM.

## 2. Analytical and experimental methods

### 2.1. Specimen-displacement 2θ correction equation

In a Debye–Scherrer diffraction experiment with a flat area detector, when a speci­men is displaced a distance *d* from its ideal position, X-rays diffracted at an angle 2θ are detected at an angle 2θ − δ. This shift in the measured angle is given by 

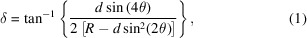

where δ is the corrective 2θ angle shift, 2θ is the angle between the incident X-rays and the detector, *R* is the speci­men-to-detector distance, and *d* is the speci­men displacement in the X-ray direction.

Equation (1) could be applied when no internal RM is included in the capillary. Generally, multiple corrections for peak shift are not used at the same time (Tsubota & Kitagawa, 2017 ▸), so if equation (1) is used, then other factors affecting peak position (King & Payzant, 2013 ▸), like zero error/zero shift (a constant value shift), axial divergence, speci­men transparency and others, should be utilized carefully or not at all. If the powder XRD pattern only covers a small range of 2θ, then these corrections to peak position could be highly corelated (Dinnebier *et al.*, 2018 ▸). Axial divergence and speci­men transparency can be better determined with peak-shape corrections (Cheary *et al.*, 2004 ▸). The derivation of equation (1) is shown in Appendix *A* [App appa]. Implementation of equation (1) in the *TOPAS* program (Bruker, 2007 ▸; Coelho, 2018 ▸) is described in Appendix *B* [App appb].

### 2.2. Implementation of speci­men-displacement corrections

The general procedure to use both the internal RM and equation (1) corrections for a speci­men displacement are the same: the displacement distance [for the equation (1) correction] or the new calibration and integration parameters (for the internal RM correction) are determined for a speci­men capillary at 25°C, and then those values are used for all higher-temperature XRD scans for that capillary location. XRD data at higher temperatures cannot be corrected individually because thermal expansion of the material also causes a shift in the Bragg peaks. The steps for each method for a series of XRD measurements at increasing temperature are described below, during which it is assumed that the capillary position does not change. Alternatively, it is possible to include speci­men displacement in a parametric refinement (Stinton & Evans, 2007 ▸), where all XRD patterns are analyzed together.

1. *Determine the speci­men displacement from the 25°C XRD scan.*

(*a*) Equation (1) correction method: add equation (1) to the Rietveld refinement of the 25°C XRD scan, fix the unit-cell parameter of the sample to its known 25°C value, and then refine the displacement value, *d*.

(*b*) Internal RM correction method: use *GSAS-II* (Toby & Von Dreele, 2013 ▸), *FIT2D* (Hammersley *et al.*, 1995 ▸; Hammersley, 2016 ▸) or another area-detector image-processing program to determine the calibration coefficients (beam center, speci­men-to-detector distance, tilt angle, tilt rotation, detector penetration) and integration coefficients (2θ limits, absorption) from the RM at 25°C.

2. *Include the corresponding 2θ correction for all other XRD scans for this speci­men capillary position.*

(*a*) Equation (1) correction method: fix the *d* value in equation (1) (turn off its refinement) to restrain its value to that of the 25°C XRD scan. Then refine the structure with all XRD scans at higher temperatures using the same *d* value.

(*b*) Internal RM correction method: use the calibration and integration parameters found previously for the 25°C scan to perform azimuthal integration of all subsequent area-detector XRD scans at higher temperatures.

### 2.3. Synchrotron experiments

Powder XRD experiments were performed to examine both correction methods at beamline 28-ID-2 (Shi *et al.*, 2013 ▸) at the National Synchrotron Light Source II (NSLS II) at Brookhaven National Laboratory (BNL) (Upton, NY, USA). The wavelength was 0.1847 Å (67.1231 keV) and the speci­men-to-detector distance was 1423.3–1426.7 mm. This range of distances corresponded to half a rotation of a misaligned capillary. Data were acquired in a 2θ range of 1–17° using a 406 mm by 406 mm (16′′ by 16′′) flat scintillator area detector. Standard reference material (SRM) 674b (NIST, Gaithersburg, MD, USA) CeO_2_ powder was used to determine calibration and integration coefficients (including the speci­men-displacement correction).

A basic Rietveld refinement (Rietveld, 1969 ▸; Loopstra & Rietveld, 1969 ▸) was performed on all data sets so as not to obscure the effect of each displacement correction. Refinements were carried out with *TOPAS* (Bruker, 2007 ▸; Coelho, 2018 ▸) in the 2θ range 2–16.5°. The speci­men-to-detector dis­tance (*R*) was determined via the internal SRM 674b CeO_2_ image calibration. A modified pseudo-Voigt function was used to model peak profiles. The starting crystal structure for CeO_2_ (Kümmerle & Heger, 1999 ▸) was refined with the Chebyshev background function (5th to 8th order as appropriate), the scale of phase(s) present and the unit-cell parameter(s) of each phase. Additional parameters (such as zero error, atomic displacement parameter or occupancy on atom sites) were not used to make the effect of the speci­men-displacement correction on the unit-cell parameters clearer.

Two experiments were used to evaluate the efficacy and demonstrate the application of the equation-based 2θ cor­rection method in equation (1). (i) The measurement of a Bragg peak shift in an XRD pattern due to a speci­men displacement was compared with the calculated shift given by equation (1). (ii) A capillary with SRM 674b CeO_2_ was displaced to several locations at which XRD scans were captured. Both the internal RM and the equation (1) correction methods were applied to each CeO_2_ XRD scan, which resulted in a measure of how the unit-cell parameters were affected by the speci­men displacements for this synchrotron experiment, as well as the accuracy and precision of the equation (1) correction method, compared with the internal RM correction method. A third experiment is shown in the supporting information, in which ZrW_2_O_8_ and 5 wt% Pt as an RM were measured to show how a small amount of internal RM affected the accuracy of each 2θ correction for a data set studied from 25 to 800°C.

## 3. Results and discussion

### 3.1. Fit of equation (1) with measured Bragg peaks

**Figure 3:**
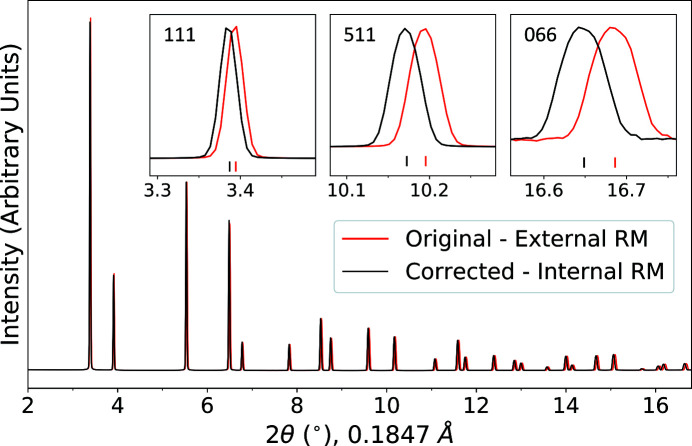
Two SRM 674b CeO_2_ XRD patterns shown with and without the 2θ shift caused by a speci­men displacement of −3.30 mm in the integration parameters when the speci­men-to-detector distance is 1426.71 mm and the wavelength is 0.1847 Å (67.1231 keV). The inset shows three reflections, *i.e.* 111, 511 and 066, in which a larger shift is seen as 2θ increases (up to 45°).

**Figure 4:**
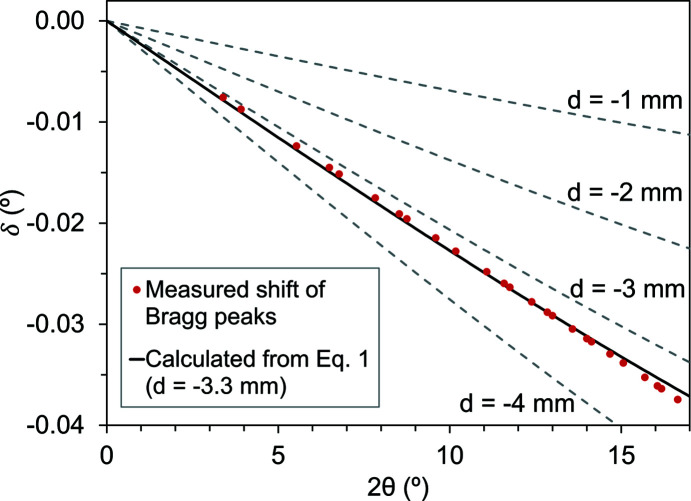
A comparison of the measured 2θ shift (δ) in the Bragg peaks for two SRM 674b CeO_2_ XRD patterns with the calculated value using equation (1), where *R* = 1426.71 mm and *d* = −3.30 mm, as determined by area-detector image calibration.

The Bragg peak shift in an SRM 674b CeO_2_ powder speci­men caused by a speci­men-displacement of −3.30 mm was measured in order to compare the values calculated from equation (1) for the same *R* and *d* values. Because CeO_2_ is a common calibrant material, new calibration and integration parameters can be determined for each movement of the capillary from the area-detector images. An XRD pattern collected on an area detector was azimuthally integrated with two sets of calibration and integration parameters. (i) Parameters derived from an area-detector image of an external CeO_2_ scan were collected at the start of the experiment with a speci­men-to-detector distance of 1423.41 (7) mm, a tilt angle of −1.498 (10)° and a tilt rotation of 5.28 (99)°. (ii) Parameters were derived from the internal CeO_2_ scan with a speci­men-to-detector distance of 1426.71 (6) mm, a tilt angle of −1.517 (9)° and a tilt rotation of 6.4 (9)°. The CeO_2_ XRD pattern is shown with and without the 2θ shift this caused in Fig. 3 ▸, where a larger shift was seen as 2θ increased, as expected from equation (1), up to 45°. Fig. 4 ▸ shows the measured 2θ shift for every Bragg peak and plots it with the expected peak shift from equation (1) for a displacement of −3.30 mm. Equation (1) describes the measured 2θ shift of Bragg peaks with good accuracy, the coefficient of determination, *R* ^2^, being 0.997.

### 3.2. Correction of the CeO_2_ unit-cell parameters

The magnitude of the change in unit-cell parameters determined by Rietveld refinement due to a speci­men displacement, *d*, depends on several factors in addition to the magnitude of *d*, including 2θ angle range, unit-cell size of the speci­men material, X-ray energy and thermal expansion. A CeO_2_ speci­men was displaced to four speci­men-to-detector distances from 1423.3 to 1426.7 mm at 25°C, where 13 XRD scans were collected to show the effect on unit-cell parameter value and determine the effectiveness of both 2θ correction methods. The 3.3 mm of speci­men-to-detector distance variability examined here was due to the direction in which a misaligned capillary holder was rotated. Each speci­men was aligned in the direction perpendicular to the X-ray direction by scanning through the X-ray beam and finding the location with maximum X-ray absorption. Because all XRD scans were collected at 25°C, it is expected that after the corrections the measured unit-cell parameter should be a constant value.

**Figure 5:**
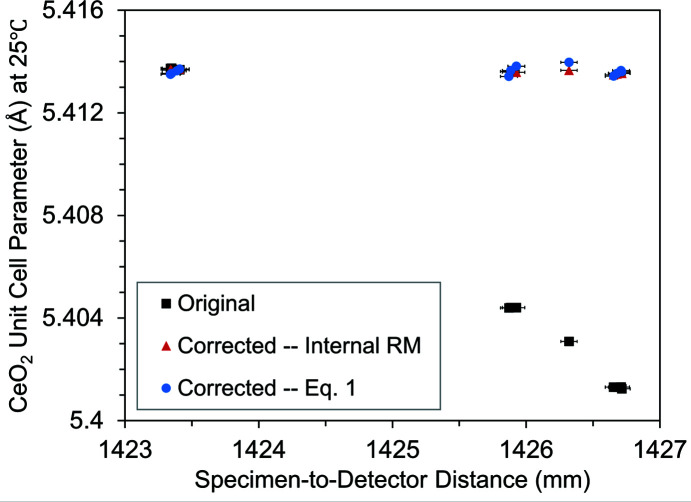
CeO_2_ unit-cell parameters at 25°C at several displacement locations with corrections from the internal reference material and equation (1). ‘Original’ used integration parameters from an external CeO_2_ XRD scan at the start of the experiment, ‘Corrected–Internal RM’ used integration parameters from the internal SRM 674b CeO_2_, and ‘Corrected–Eq. 1’ used equation (1) during the Rietveld refinement step. Both corrections were accurate, but the internal RM correction had higher precision than equation (1).

Fig. 5 ▸ compares the CeO_2_ unit-cell parameters, including the uncorrected original data (determined with integration parameters from an external RM), data corrected by equation (1) and data corrected by new integration parameters with the internal RM. The standard deviation of the unit-cell parameter corrected by equation (1) was about 36 times smaller than that of the original uncorrected data, and about two times larger than that of the RM-based correction. It was expected that the internal RM correction method would have the highest precision because it could also take into account any change in the beam center, tilt angle, tilt rotation, detector penetration and absorption, whereas equation (1) corrected only the speci­men-to-detector distance. However, the comparison provided a good measure of the accuracy of the correction by equation (1), which showed excellent agreement between the two methods. Tabulated values are listed in Section S2 of the supporting information.

Comparison of the *R* factors (*R*_wp_, GoF *etc*.) for Rietveld refinement fits were less useful in checking the effectiveness of the equation (1) correction method than the deviation of unit-cell parameter values from the expected value at 25°C in Fig. 5 ▸. During Rietveld refinements, improvements in the *R* factors meant there was a closer fit of the structural model with the experimental data, but if the model was less realistic chemically or physically then it should not be used. This was indeed the case here; when equation (1) was not used, then the 2θ shift from speci­men displacement could be compensated for with a change in unit-cell size to give nearly identical *R* factor values between each model, but this led to a unit-cell parameter that was not realistic for CeO_2_ at 25°C. It is important for researchers to consider chemical plausibility and not rely only on metrics described by *R* factors (Toby, 2006 ▸). Rietveld refinement figures and corresponding tables of crystallographic and experimental information are shown in Section S1 of the supporting information. The supporting information includes hyperlinks to an online repository of area-detector image files (.tif f) and integrated XRD files (.xye) that were analyzed for this study.

## 4. Conclusion

It is well known that internal RMs (CeO_2_, LaB_6_, Si, Al_2_O_3_, SiO_2_ *etc*.) can be used as calibrants for powder XRD when accurate unit-cell parameters are required. However, it is common in the literature to perform one full detector calibration with an external RM collected at the start of the experiment, instead of with an internal RM mixed with each speci­men. This study compared the correction from an internal RM with a novel correction equation in determining the effect of a 3.3 mm speci­men-to-detector displacement on the CeO_2_ unit-cell parameters. Equation (1) was proposed to correct for 2θ shifts caused by speci­men displacements in Debye–Scherrer XRD experiments using a flat area detector. This equation does not require an internal RM and is applied during the Rietveld refinement step, so that new integration parameters from 2D area-detector images are not needed for each speci­men. This would allow a full detector calibration at the start of an experiment, and then the use of equation (1) for small misalignments in speci­men-to-detector distance that occur throughout an experiment, instead of a new image calibration and reintegration for each speci­men capillary. Equation (1) is analogous to existing speci­men-displacement correction equa­tions for other experimental geometries (Debye–Scherrer geometry with a curved area detector and Bragg–Brentano geometry), but it has a different functional form and is for an experimental setup that is commonly used in syn­chro­tron powder XRD experiments.

## 5. Related literature

The following references are cited in the supporting information: Mary *et al.* (1996 ▸); Owen & Yates (1934 ▸); Sarin *et al.* (2006 ▸); Touloukian (1975 ▸).

## Supplementary Material

The area detector image files (.tiff) and integrated XRD files (.xye) analyzed in this manuscript can be downloaded from an online repository.: https://doi.org/10.5281/zenodo.7015931

Rietveld refinement figures and corresponding tables; tabulated values for the agreement between the two methods; details of a third experiment. DOI: 10.1107/S1600576722011360/vb5047sup1.pdf

## Figures and Tables

**Figure 6:**
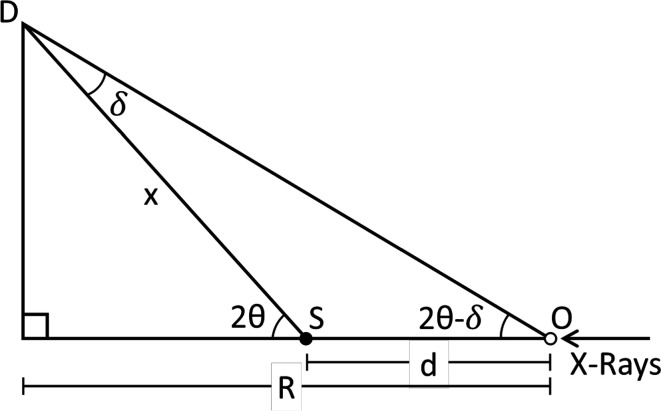
Ray diagram of a Debye–Scherrer XRD experiment showing the effect of speci­men displacement on 2θ value. The speci­men capillary is at S, the idealized speci­men position (typically the goniometer center) is at O, which is a length *R* from the detector, length *d* becomes more positive as S moves towards the detector, and X-rays are traveling from right to left. X-rays are diffracted through an angle 2θ at S, hitting the detector at D, which are measured at an angle 2θ − δ.

## Appendix A Equation (1) derivation (see Fig. 6 ▸)

Starting from the law of sines:

## Appendix B Implementation in Bruker *TOPAS* software

Equation (1) can be used during peak fitting in *TOPAS* (Bruker, 2007 ▸; Coelho, 2018 ▸) by adding the lines of code shown below to an input (.inp) file. Th, Rs, Rad and th2_of fset are global variables already defined in the *TOPAS* scripting language which stand for theta (half of the 2θ value), secondary radius (the distance from speci­men to detector), declaration of the variable as type radians and the shift of the data in the *x*-ordinate variable (commonly in degrees for the 2θ angle), respectively. Equation (1) could also be programmed as a *TOPAS* macro which would be added to the local.inc file so it does not need to be defined in each input file. Descriptions for implementing related methods by *TOPAS* macro were given elsewhere (Evans, 2010 ▸; Rowles & Buckley, 2017 ▸; Gozzo *et al.*, 2010 ▸; Dinnebier *et al.*, 2018 ▸; Coelho *et al.*, 2011 ▸) and are in the *TOPAS* technical reference.

Rs 1423

prm d 0.0 min -5 max 5

th2_of fset = Rad ArcTan((d Sin(4 T h)) / (2 (Rs - d Sin(2 T h)^2)));
