# Specimen-displacement correction for powder X-ray diffraction in Debye–Scherrer geometry with a flat area detector. Erratum

**DOI:** 10.1107/S160057672300153X

**Published:** 2023-03-21

**Authors:** Benjamin S. Hulbert, Waltraud M. Kriven

**Affiliations:** a Materials Science and Engineering, University of Illinois at Urbana-Champaign, 1304 W. Green St., Urbana, Illinois 61801, USA; Australian Synchrotron, ANSTO, Australia

**Keywords:** Debye–Scherrer, transmission, speci­men-to-detector distance, displacement correction equation, powder X-ray diffraction, area detectors

## Abstract

An error in Fig. 2(*b*) in the paper by Hulbert & Kriven [*J. Appl. Cryst.* (2023), **56**, 160–166] is corrected.

In the article by Hulbert & Kriven (2023 ▸), there is an error in Fig. 2(*b*) which shows the Bragg–Brentano geometry for an X-ray diffraction (XRD) experiment. The arc denoting the angle 2θ + δ was mistakenly drawn so that it ended at the base of the specimen. However, it should extend to the incident beam. The revised Fig. 2(*b*) diagram is given here, shown in Fig. 1 ▸. Both the derived equation and the conclusions in the original article are unaffected by this figure correction.

## Figures and Tables

**Figure 1:**
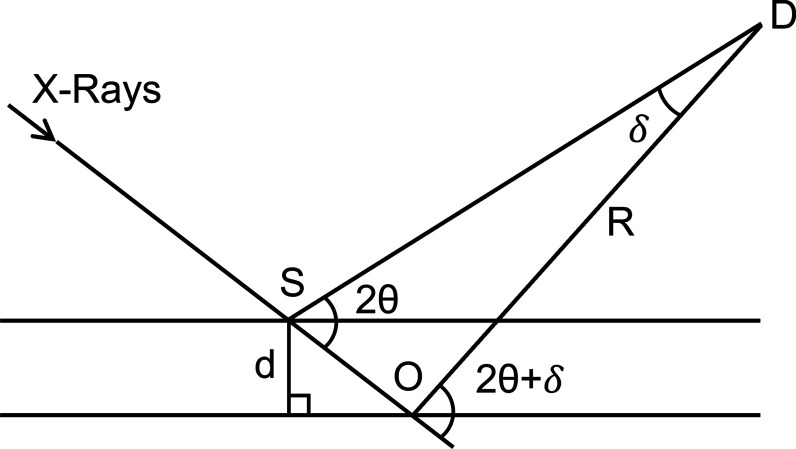
Revised diagram showing Bragg–Brentano XRD geometry. The original figure (Hulbert & Kriven, 2023 ▸) showed the angle 2θ + δ drawn incorrectly.
