# Supplementary material for: Specimen-displacement correction for powder X-ray diffraction in Debye–Scherrer geometry with a flat area detector
Source: J Appl Crystallogr. 2023 Feb 1;56(Pt 1):160–6. doi: 10.1107/S1600576722011360 (PMC9901925; doi:10.1107/S1600576722011360)
Supplement: Supplementary file 1 [file j-56-00160-sup1.pdf]

JOURNAL OF
APPLIED
CRYSTALLOGRAPHY

Volume 56 (2023)

Supporting information for article:

Specimen-displacement correction for powder X-ray diffraction in Debye–Scherrer geometry with a flat area detector

Benjamin S. Hulbert and Waltraud M. Kriven

The following is supporting information for the manuscript: Specimen displacement correction for X-ray diffraction in Debye-Scherrer geometry with flat area detector.

The area detector image files (.tiff) and integrated XRD files (.xye) analyzed in this manuscript can be downloaded from an online repository: https://doi.org/10.5281/zenodo.7015931

Contents:

S1. CeO_2 Rietveld Fit
S2. CeO2 Unit-cell Parameters and Specimen-to-detector Distances
S3. ZrW2O8 Temperature Series Correction
S4. Zr_2O_8 Unit-cell Parameters and Specimen-to-detector Distances

S1. CeO_2 Rietveld Fit

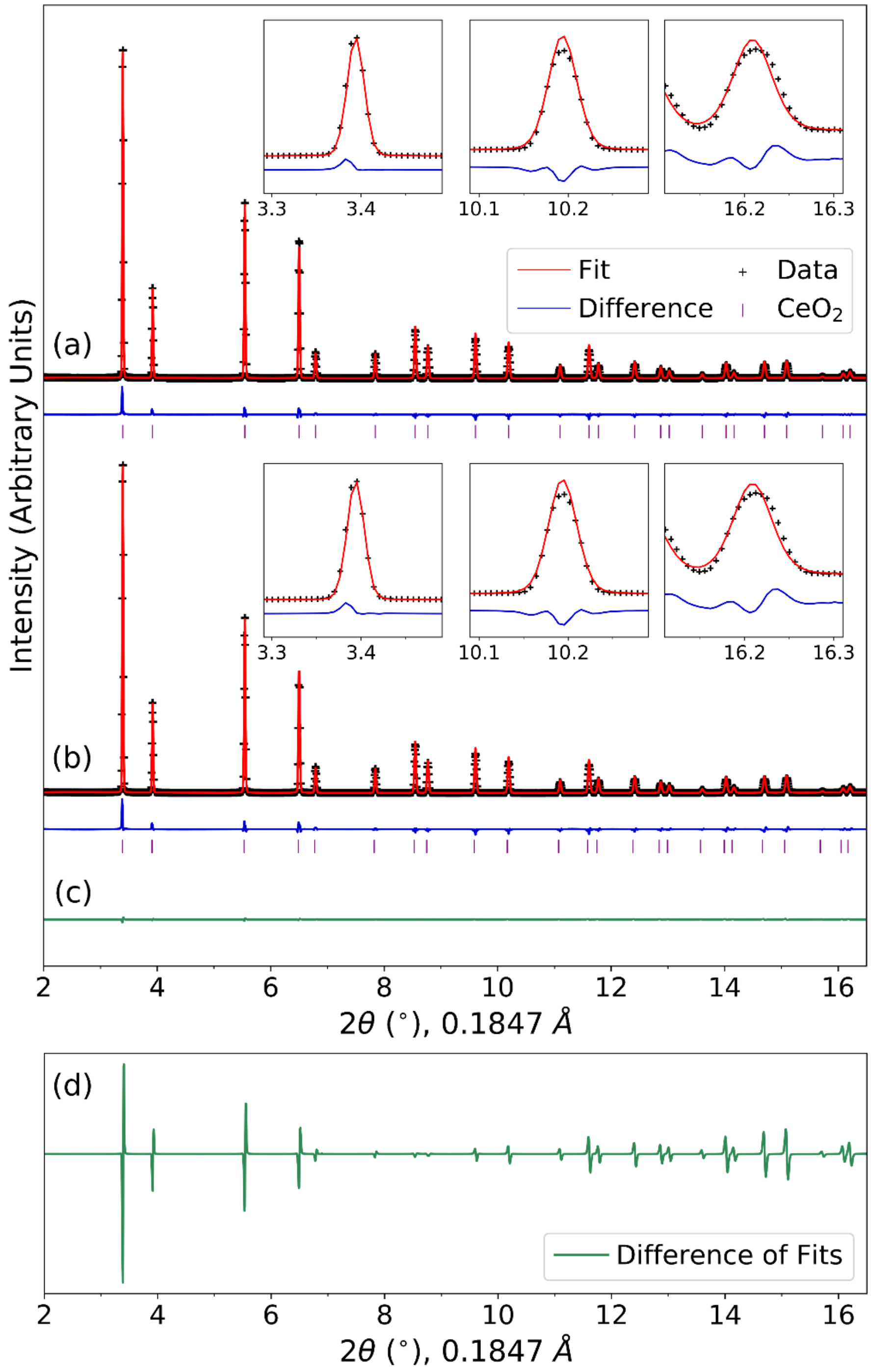

Figure S1 Rietveld refinement of the CeO_2 structure at 25 °C for the (a) uncorrected and (b) Eq. 1 corrected XRD data using TOPAS. The difference between the Rieveld fits for the uncorrected and Eq 1-corrected refinements are displayed in (c) using the same scale as (a) and (b) and rescaled in (d) to show its features in detail. This difference in Rietveld fits is nearly zero because the shift in peaks is compensated in (a) with the unit-cell parameter and in (b) by the d-value in Eq. 1. Details of the refinement are given in Table S1.

Table S1 Crystallographic and Rietveld data for the CeO_2 dataset shown in Fig. S1 with and without Eq. 1. Differences are shown in bold.

	**CeO_2 – Uncorrected**	**CeO_2 – Corrected by Eq. 1**
Crystal Data		
Crystal System	Cubic	
Space group (number)	$Fd\bar{3}m$ (225)	
Z	4	
T (°C)	25	
**a, b, c (Å)**	**5.40132(2)**	**5.41368(2)**
α, β, γ (º)	90	
Unit-Cell Volume (Å^3)	**157.5792(19)**	**158.664(2)**
Rietveld Refinement		
Computer Program	TOPAS	
Refined variables	CeO_2 unit-cell parameter, phase scale, background	CeO_2 unit-cell parameter, phase scale, background, **d (specimen displacement)**
Specimen displacement (mm), d	**Not Determined**	**-3.31**
Displacement Correction Method	**None**	**Eq. 1**
R_{wp} (%)[b]	**5.14**	**5.47**
R_{exp} (%)[b]	0.34	0.34
R_p (%)[b]	**3.44**	**3.60**
GoF (%)[b]	**14.89**	**15.85**
R_{Bragg}[b]	**3.86**	**3.87**

[b]Values are as defined in Bruker TOPAS Software

S2. CeO_2 Unit-cell Parameters and Specimen-to-detector Distances

Table S2 CeO_2 unit-cell parameter averages from original, internal standard reference material correction, and Eq. 1 correction methods shown in Fig. 5.

Specimen-to-Detector Distance		Original -- Integration parameters from external RM		Corrected -- Integration parameters from internal RM		Corrected -- Eq. 1 during Rietveld Refinement	
Distance (mm)	ESD	CeO_2 Unit-Cell Parameter (Å)	ESD	CeO2 Unit-Cell Parameter (Å)	ESD	CeO2 Unit-Cell Parameter (Å)	ESD
1423.41	0.07	5.41368	0.00003	5.41370	0.00003	5.41371	0.00003
1423.35	0.07	5.41374	0.00003	5.41367	0.00003	5.41354	0.00003
1423.39	0.07	5.41369	0.00003	5.41370	0.00003	5.41364	0.00003
1426.32	0.06	5.40309	0.00003	5.41366	0.00002	5.41397	0.00003
1426.71	0.06	5.40131	0.00004	5.41355	0.00002	5.41365	0.00004
1426.65	0.06	5.40130	0.00004	5.41348	0.00002	5.41343	0.00004
1426.68	0.06	5.40130	0.00004	5.41352	0.00002	5.41353	0.00004
1426.68	0.06	5.40131	0.00004	5.41354	0.00002	5.41352	0.00004
1426.72	0.06	5.40124	0.00004	5.41354	0.00002	5.41360	0.00004
1425.87	0.06	5.40440	0.00002	5.41361	0.00002	5.41342	0.00003
1425.93	0.06	5.40440	0.00002	5.41358	0.00002	5.41381	0.00003
1425.89	0.06	5.40440	0.00002	5.41359	0.00002	5.41365	0.00003
1423.34	0.07	5.41373	0.00003	5.41371	0.00003	5.41350	0.00003

S3. ZrW_2O_8 Temperature Series Correction

S3.1. ZrW_2O_8 Experimental Methods

For the ZrW_2O_8 specimens, Pt was an internal reference material (RM) (Touloukian, 1975) and a thermal conductor. The high temperatures for the ZrW_2O_8 specimens were achieved with a hexapole lamp optical furnace at beamline 28-ID-2 at NSLS II. The ZrW_2O_8 specimen data measured with a specimen spinner was collected at beamline 17 BM-B at the Advanced Photon Source (APS) at Argonne National Laboratory (ANL) with a wavelength of 0.24117 Å (51.4062 KeV), specimen-to-detector distance of 1001.8 mm was determined from a single LaB_6 image calibration, a 432 mm by 432 mm (17’’ by 17”) Varex 4343CT flat area detector, and quadrupole lamp optical furnace (Sarin *et al.*, 2006).

A basic Rietveld refinement as described in §2.3 was performed on all datasets so as not to obscure the effect of each displacement correction. Starting crystal structures for Pt (Owen and Yates, 1934), and ZrW_2O_8 (Mary *et al.*, 1996) were refined with the Chebyshev background function (5th to 8th order as appropriate), scale of phase(s) present, and unit-cell parameter(s) of each phase.

S3.2. ZrW_2O_8 Displacement Correction

Negative thermal expansions in ZrW_2O_8 unit-cell parameters are plotted in Fig. S2, which show that the original integration parameters which included the specimen displacement error, both types of analytical specimen displacement corrections, as well as data from a new specimen capillary collected with a specimen spinner to keep the capillary in the center of rotation. Corrections from Eq. 1 and from the internal RM methods both yielded improvements, however the correction with internal RM was closer to the values determined with the specimen spinner.

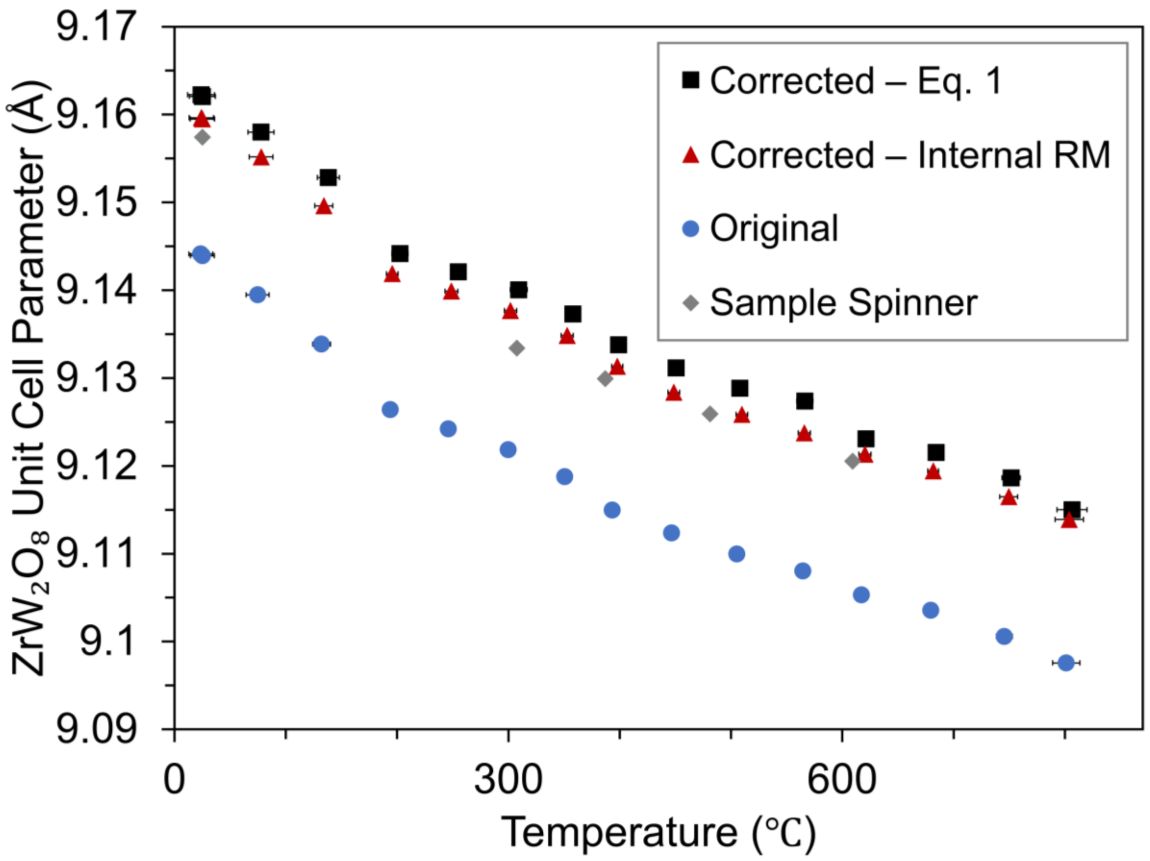

Figure S2 ZrW_2O_8 unit-cell parameters from 25 °C to 800 °C are plotted for a specimen displaced by -2.42 mm from the specimen-to-detector distance of 1426.28 mm used for area detector image integration, 'Original'. This is compared with a correction based on new integration parameters from an internal Pt reference material, 'Corrected – Internal RM,' and the Eq. 1 correction during the Rietveld refinement step, 'Corrected – Eq. 1'. This is compared with a dataset collected from a specimen centered by rotating the specimen capillary, 'Specimen Spinner,' for which the specimen displacement is assumed to be negligible.

Both correction methods were more accurate for pure CeO_2 than for ZrW_2O_8. This decrease in accuracy was likely due to the fact that the ZrW_2O_8 specimen was composed of only 5 wt% Pt RM and because of overlap of the Pt Bragg peaks with the ZrW_2O_8 Bragg peaks. The variation in intensity between the CeO_2 rings and the surrounding background was greater than that of Pt and the surrounding background which also included ZrW_2O_8 peaks, making the precise location of each ring less certain.

S4. ZrW_2O_8 Bragg Peak Shift

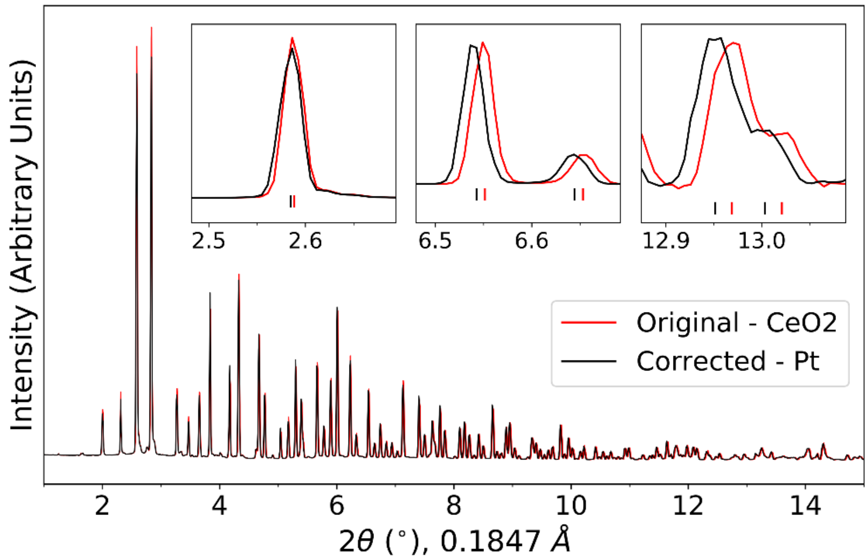

Figure S3 Two ZrW_2O_8 XRD patterns shown with and without the 2θ shift caused by a specimen displacement of -2.42 mm when the specimen-to-detector distance is 1423.41 mm. A larger shift is seen as 2θ increases (up to 45º).

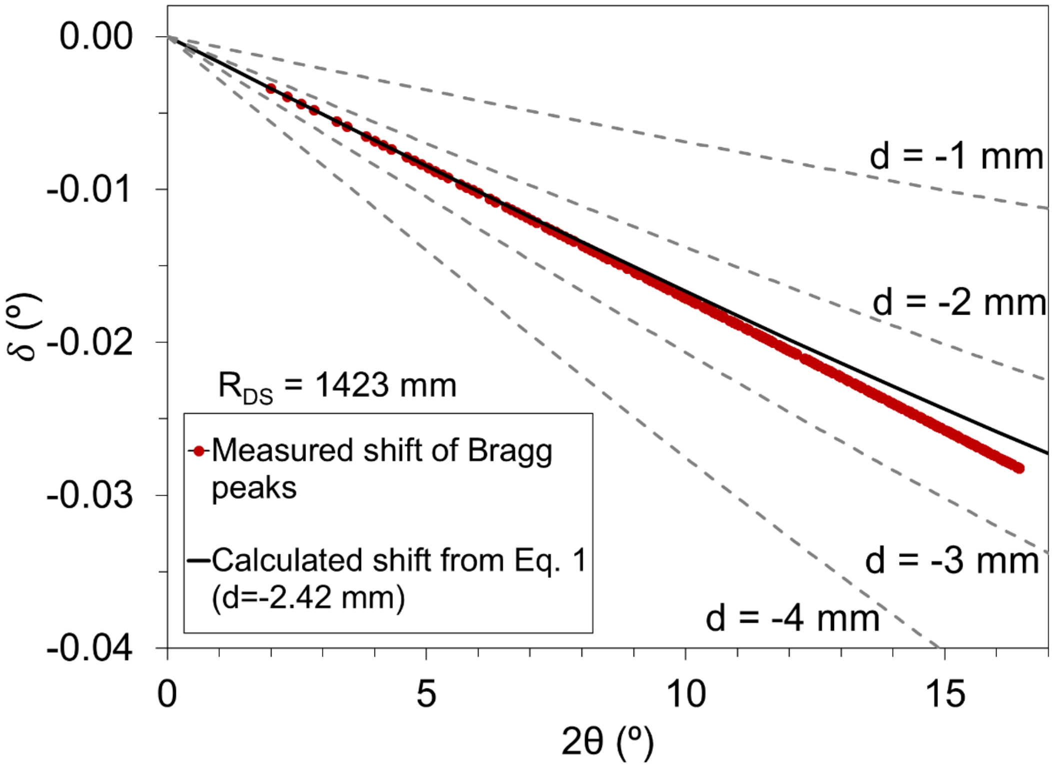

Figure S4 A comparison of measured 2θ shift in the Bragg peaks shown in the XRD patterns with the calculated 2θ using the proposed Eq. 1 where R_{DS} = 1425.9 mm and d = -2.42 mm.

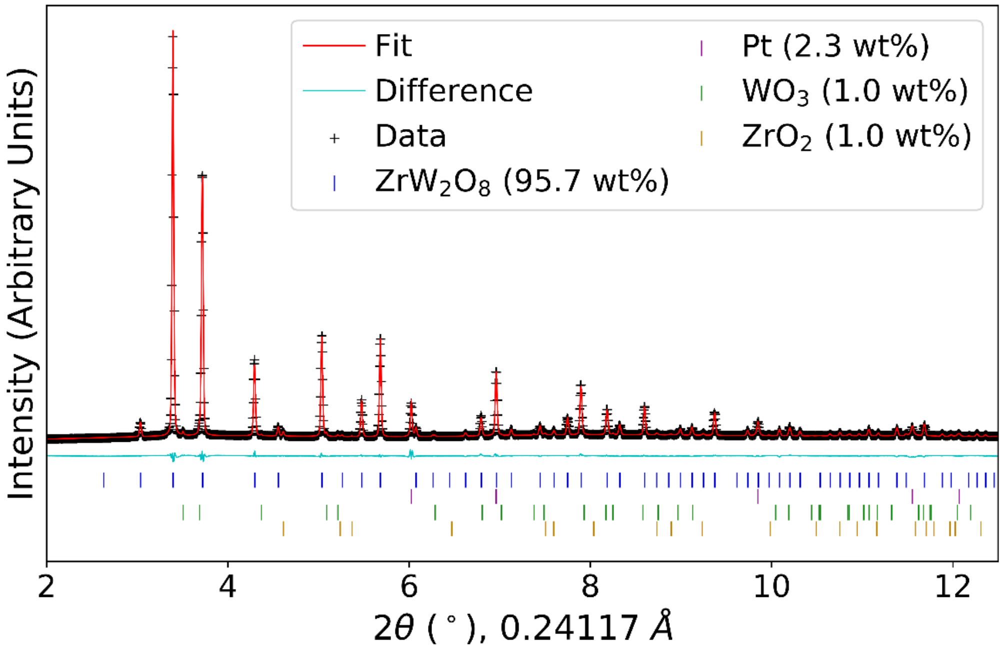

Figure S5 Rietveld refinement of the ZrW_2O_8 structure at 1210 °C. Diffraction peaks from ZrW_2O_8 and minor phases of WO_3 and ZrO_2 overlap with some of the reference material Pt peaks.
